# Focus on Marine Animal Safety and Marine Bioresources in Response to the SARS-CoV-2 Crisis

**DOI:** 10.3390/ijms232315136

**Published:** 2022-12-01

**Authors:** Yao Yang, Jiacheng Li, Fang Han

**Affiliations:** 1 Fisheries College, Jimei University, Xiamen 361021, China; 2 Key Laboratory of Healthy Mariculture for the East China Sea, Ministry of Agriculture and Rural, Xiamen 361021, China; 3 Affairs Fujian Provincial Key Laboratory of Marine Fishery Resources and Eco-Environment, Xiamen 361021, China

**Keywords:** marine metabolites, anti-SARS-CoV-2, COVID-19, drug development, marine mammals

## Abstract

SARS-CoV-2 as a zoonotic virus has significantly affected daily life and social behavior since its outbreak in late 2019. The concerns over its transmission through different media directly or indirectly have evoked great attention about the survival of SARS-CoV-2 virions in the environment and its potential infection of other animals. To evaluate the risk of infection by SARS-CoV-2 and to counteract the COVID-19 disease, extensive studies have been performed to understand SARS-CoV-2 biogenesis and its pathogenesis. This review mainly focuses on the molecular architecture of SARS-CoV-2, its potential for infecting marine animals, and the prospect of drug discovery using marine natural products to combat SARS-CoV-2. The main purposes of this review are to piece together progress in SARS-CoV-2 functional genomic studies and antiviral drug development, and to raise our awareness of marine animal safety on exposure to SARS-CoV-2.

## 1. Introduction

There are some common human coronaviruses which people normally get infected with, such as 229E, NL63, OC43, and HKU1 according to a report of the Center for Disease Control and Prevention (CDC) of the US (cdc.gov). Recently, some zoonotic coronaviruses have transmitted to, and attacked, humans regionally and globally, developing into human coronaviruses exemplified by the three viruses MERS-CoV (the β-coronavirus causing Middle East respiratory syndrome; MERS), SARS-CoV (the β-coronavirus causing severe acute respiratory syndrome; SARS), and SARS-CoV-2 (the novel coronavirus causing coronavirus disease; COVID-19). The burst of the infection by the latest coronavirus in humans was first reported in Wuhan, Hubei, China in December 2019, with the interim name of coronavirus 2019-nCoV proposed by the World Health Organization (WHO). Shortly after, a Public Health Emergency of International Concern (PHEIC) was declared by WHO on 30 January 2020 [1]. Later, the novel pathogenic coronavirus was officially renamed SARS-CoV-2 by the International Committee on Taxonomy of Virus (ICTV), and the disease was named COVID-19 (coronavirus disease 2019) in the system of International Classification of Disease (ICD) by WHO on 11 February 2020 [2]. The rapid spread of SARS-CoV-2, and the sharp increase in COVID-19 cases since its onset, drew international attention and constituted a global pandemic that was announced by WHO on 11 March 2020 [3].

To mitigate the transmission of SARS-CoV-2, strict restrictions and preventive measures have been implemented, causing tremendous impact on the economics, social behavior, and many aspects of daily life. Although diverse countermeasures were imposed, the COVID-19 pandemic has still caused more than 6 million deaths globally as of the preparation of this manuscript. In addition, the SARS-CoV-2 virus has been reported to have the potential to infect animals with close contact with humans, and some wild animals as well. Tremendous efforts and funds have been invested on clinical diagnostics, research, and medicine and vaccine development to understand and control COVID-19 disease. In this review, our focuses are centered on the genomic structure of SARS-CoV-2 and their functions, its potential targeting of marine animals, and marine bioactive compounds with therapeutic effect against COVID-19.

## 2. Genomic Structure of SARS-CoV-2 and Their Functions

Coronaviruses are enveloped viruses containing a positive-sense single-stranded RNA (+ssRNA) genome, belonging to the subfamily *Coronavirinae* in *Coronaviridae* family [4]. There are four genera of coronaviruses including α, β, γ, and δ coronavirus with some specific mutations and recombination. Currently, only the α- and β- coronaviruses have been reported to infect humans, such as 229E and NL63 of α-coronavirus and OC43 and HKU1 of β-coronavirus, while the γ- and δ- coronaviruses are known to infect only birds or birds and non-human mammals, respectively [5]. The SARS-CoV-2 virus is a member of β-coronaviruses that include at least four lineages of A, B, C, and D. The SARS-CoV-2 and SARS-CoV (or SARS-CoV-1) belong to lineage B, while OC43 and HKU1 belong to lineage A and MERS-CoV belongs to lineage C [6].

### 2.1. Full Genetic Makeup of SARS-CoV-2

**Figure 1:**
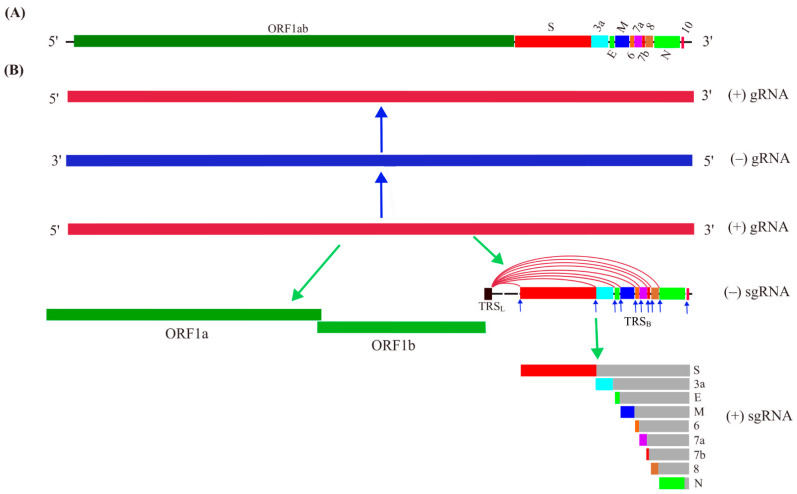
Schematic view of SARS-CoV-2 genome and its replication and transcription. (**A**) Genome structure of SARS-CoV-2. (**B**) SARS-CoV-2 genome replication and expression. The positive-sense single stranded RNA of SARS-CoV-2 can act as template guiding the synthesis of negative-sense gRNA for genome replication to (+) gRNA. The (+) gRNA can serve as mRNA for translation of the long ORF1a and ORF1ab at its 5′-terminus of genome, and the transcription of the 3′-terminus of its genome is achieved by the discontinuous synthesis of a set of subgenomic mRNAs (sgRNA) through a template switch mechanism by jumping from the transcription regulatory sequence in the genome body (TRS_B_; indicated by arrows) to the leader TRS (TRS_L_). The sgRNAs guide the synthesis of structural proteins and some accessory proteins.

After the initial clinical diagnosis of the novel coronavirus-caused disease, scientists are striving to dissect the genetic elements of SARS-CoV-2. On 3 February 2020, the full genome sequence of SARS-CoV-2 was published, which contains 29,903 nucleotides consisting of at least 14 ORFs (1ab, S, 3a, 3b, E, M, 6, 7a, 7b, 8, 9a, 9b, N, 10) with some overlaps encoding some structural, nonstructural, and accessory proteins [7]. Transcriptome data support the transcription of ORF1a, 1b, S, 3a, E, M, 6, 7a, 7b, 8, N [8]. Comparative genomic analysis further concluded that the protein-coding ORFs in the genome of SARS-CoV-2 include ORF1a, 1ab, S, 3a, 3c, E, M, 6, 7a, 7b, 8, N, and 9b [9]. Some disagreement and ambiguity exist for the numbers and names of the ORFs, such as ORF9b and 9c being also called ORF9a and 9b or ORF13 and ORF14, respectively [7,9,10,11]. Collectively, the primary genome structure of SARS-CoV-2 (Figure 1A) and its proposed protein-coding ORFs (Figure 1B) in the linear structure have been elucidated.

(+) Genomic RNA can function as mRNA directing translation into viral proteins, and can also be used as a template for the synthesis of (−) genomic RNA, which then guides new (+) genomic RNA synthesis by the action of RNA-dependent RNA polymerase (RdRp) during replication (Figure 1B). It is a common strategy for the (+) ssRNA virus to synthesize a set of subgenomic RNAs (sgRNAs) to direct the translation of its 3ʹ-proximal genes [12]. The (+) genomic RNA of SARS-CoV-2 can guide the synthesis of a polyprotein, namely pp1ab, by translating the ORF1ab located in the 5ʹ-end covering about two-thirds of the genome. The ORF1ab can also be referred to as ORF1a and ORF1b, in which ORF1a can be translated into pp1a by canonical translation, while a programmed −1 ribosomal frameshift (−1 PRF) at the end of ORF1a may happen leading to the synthesis of pp1ab by non-canonical translation (Figure 1B). The rest, approximately one-third, of the genome located at the 3′-end is transcribed into sgRNAs with varying lengths for their translation into the structural proteins S (spike), E (envelop), M (membrane), and N (nucleocapsid), and the at least six accessory proteins including 3a, 6, 7a, 7b, 8, and 9b [13]. Some ORFs, including ORF3b, 9c, and 10, obtained by computational analysis, may not encode a protein in the virus, although some studies on their ectopic expression have shown their biological functions [9,13]. One of the mechanisms proposed for the production of sgRNAs is a template switch, or jumping through the long-range base paring between the transcription regulatory sequence (ACGAAC; TRS) located in the 5′-leader (TRS_L_) and the one in the genome body (TRS_B_) before the ORFs during the synthesis of (−) gRNA. sgRNAs appear polycistronic, while it is assumed that only the first ORF after the junction part of the TRS sites is translated, and the sgRNA is named with the first ORF accordingly [14]. The transcription and translation of the gRNA and sgRNA are achieved through the action of replication and transcription complex (RTC) consisting of RdRp and other RNA processing enzymes in the double membrane vesicles (DMV) formed through transmembrane proteins nsp3/nsp4/nsp6-induced ER membrane remodeling [13,15,16].

### 2.2. ORF1ab Cleavage

**Figure 2:**
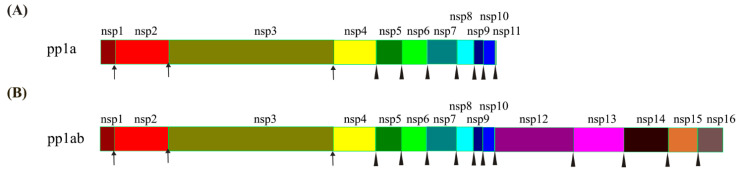
Cleavage of pp1a and pp1ab by PLpro and Mpro. (**A**) Composition and cleavage of pp1a. (**B**) Composition and cleavage of pp1ab. Arrows represent the cleavages by PLpro, and triangles represent the cleavages by Mpro. The polyprotein pp1a is cleaved by PLpro and Mpro into 11 non-structural proteins (nsps) after its synthesis at their respective sites. The pp1ab is synthesized by translating the ORF1ab with the programmed −1 ribosomal frameshift (−1 PRF) on nsp11 and cleaved by PLpro and Mpro into 15 nsps.

ORF1ab encodes 16 non-structural proteins (nsps) with pp1a being cleaved into nsp1-11 and pp1ab into nsp1-10 and nsp12-16 [9] for the initial hijacking and colonization of host cells [17]. The cleavage of pp1a and pp1ab are mediated by two proteases, nsp3 (papain-like protease, PLpro) and nsp5 (chymotrypsin-like protease, 3CLpro, also called main protease, Mpro) [18]. The recognition sites for PLpro and Mpro are LXGG↓XX and X-(L/F/M)-Q↓(G/A/S)-X, respectively, and PLpro is responsible for the first three cuts to release nsp1/nsp2/nsp3, while Mpro cleaves the rest of the sites in pp1a/pp1ab after nsp4 [19] (Figure 2).

ORF1ab encodes components to facilitate SARS-CoV-2 immediate infection (Table 1). nsp1 binds to the small subunit of the host cell ribosome to block the translation of host transcripts while initiating the translation of the viral genome [20,21]. nsp2 is a zinc-finger protein that can interact with the human 4EHP-GIGYF2 complex to impact the functioning of post-transcriptional silencing machinery to suppress the host defense response [22]. nsp3, the longest nsp in the genome, can interconnect with the host proteins involved in the immune response, RNA metabolism, and some fundamental cellular functions, suggesting the attack of host cell functioning by nsp3 [23]. The nsp7-nsp8 tetramer (dimer of dimer) acts as the primase to initiate RNA replication and interact with nsp12 to form a replication/transcription complex [24]. nsp12 encodes RNA-dependent RNA polymerase responsible for viral replication and transcription with the participation of helicase encoded by nsp13 [25]. nsp14 is a bifunctional protein which possesses an N^7^-methyltransferase activity at its C-terminus and 3ʹ-5ʹ exonuclease (ExoN) activity at its N-terminus. The nsp14 interacts with nsp10 cofactor to form a complex to excise its proofreading during the RNA synthesis mediated by the RdRp, which lacks extension fidelity [26]. In addition, the nsp14-nsp10 complex catalyzes the formation of cap-0 structure (m7GpppA) of the newly synthesized viral RNA. Furthermore, the nsp16 methyltransferase and the activation cofactor nsp10 work as a complex to methylate the cap-0 structure at 2′-O position to form the cap-1 structure (m^7^G_ppp_A_m_) [27]. Accumulating studies have shown that the nsps processed from ORF1ab can suppress the innate immunity of host cells to evade the host defense response, such as the suppression of type I interferon production [28].

### 2.3. Structural Proteins

**Figure 3:**
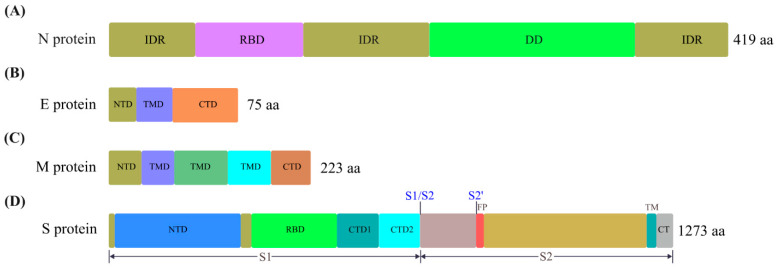
Domain structures of SARS-CoV-2 N, E, M, and S structural proteins. (**A**) N protein. IDR, intrinsically disordered region. RBD, RNA binding domain. DD, dimerization domain. (**B**) E protein. NTD, N-terminal domain (NTD). TMD, transmembrane domain. CTD, C-terminal domain. (**C**) M protein. (**D**) S protein. RBD, receptor-binding domain. FP, fusion peptide. TM, transmembrane anchor. CT, cytoplasmic tail. The aa denotes the amino acids. The size of the diagram of S protein is not proportional to those of N, E, and M proteins.

The structural proteins of SARS-CoV-2 include S, E, M, and N proteins, and their structural domains are shown in Figure 3. The SARS-CoV-2 virion contains the gRNA bound with N protein packed within a capsid and the M, E, and S proteins incorporated in the outside membrane.

#### 2.3.1. N Protein

The N protein consists of two structural domains and three intrinsically disordered regions (IDRs). The N-terminal domain (NTD) and the C-terminal domain (CTD) are flanked with IDRs at the N-terminus and C-terminus, respectively, with the central IDR in between [30]. The two structure domains are also called the RNA-binding domain (RBD) and dimerization domain (DD), and the three IDRs are named NTD, a central linker (LINK), and CTD, respectively (Figure 3A) [31]. The structural signatures of N protein suggest that the N protein can physically interact with the gRNA of SARS-CoV-2 for packaging through liquid-liquid phase separation (LLPS) to form a condensate, and this process is proposed to be driven by the recognition and binding of a specific element in the 5ʹ-end of ORF1ab as virion assembly excludes the sgRNAs [32]. Functional domain analysis suggests that the L/Q-rich subdomain of the central IDR or central linker of N protein is essential for its interaction with gRNA [30], while the other domains are also important for their interaction, as their presence can compensate for the missing of SR-rich or L/Q-rich region of the central IDR [30,33]. Further probing into the cis-acting element in the ORF1ab region for N protein-mediated gRNA packaging shows that the core element at 20080-21171 nt (nsp15-nsp16 region) is sufficient for viral packaging [34]. The packaging process proposed is that the N protein binds to the packaging signal in the gRNA to initiate the condensation and recruit more N protein along the gRNA. The recruited N proteins can interact through the dimerization domain to form large oligomers, eventually forming the mature and stable condensate of viral RNA-protein complex [30,31].

#### 2.3.2. E Protein

The E protein is the smallest protein of SARS-CoV-2 structural proteins, with only one transmembrane domain (TMD) (Figure 3B) [35]. During viral infection, a small portion of the expressed E protein is incorporated into the viral envelope while the majority is in the ER-Golgi intermediate compartment (ERGIC) of the host cell, assembling into a pentamer imbedded in the membranes as ion channels (ICs). The C-terminal domain of the E protein faces the cytoplasmic side, and the N-terminal domain faces the ER-lumen [36]. The insertion of the E protein in the ER, Golgi, and ERGIC membranes can induce membrane curvature towards the cytoplasm, potentially facilitating the budding of the SARS-CoV-2 virions. In addition, the channel-like structure formation in the ER/Golgi membrane system can stimulate viral entry and trafficking in the host cell and affect the normal physiological activity of the host cell by altering membrane permeability [35].

#### 2.3.3. M Protein

The M protein is the most abundant structural protein of SARS-CoV-2, consisting of three transmembrane domains (TMD) flanked with a short N-terminal domain (NTD) and a β-sheet sandwich domain (BD or CTD) at the C-terminus (Figure 3C). The M protein is also located in the ERGIC, where it can form a dimer with some structural plasticity, while it is unlikely to function as a channel for ion conduction [37]. The M protein is considered the major driver for virial assembly, which is supported by the observation that it can interact with the N protein and ribonucleoprotein complex, further recruiting S and E proteins, potentially through physical interactions to form virion particles [37,38].

#### 2.3.4. S Protein

The S protein is the most extensively studied structural protein. It is a type I membrane protein with only a single-span transmembrane domain, while it is assembled as a trimer anchored in the surface of the SARS-CoV-2 virion [39]. Basically, the S protein contains a short signal peptide located at the N-terminus, followed by S1 and S2 subunits in the middle and at the C-terminus, respectively (Figure 3D). The S1 and S2 subunits can be further divided into several different domains [40]. The S1 subunit contains an N-terminal domain (NTD), a receptor-binding domain (RBD), and two C-terminal domains (CTD1 and CTD2). The RBD harbors the receptor-binding motif (RBM) interacting with the ACE2 receptor on the host cell [41]. The S2 subunit contains a fusion peptide (FP), transmembrane anchor (TM), cytoplasmic tail (CT), and other domains [40,42]. During SARS-CoV-2 virus maturation in the infected host cells, the connection between S1 and S2 subunits is cleaved by furin protease, and then the S1 and S2 subunits are linked non-covalently. The cleavage of S1 and S2 by furin definitely promotes viral infection, but other protease may also function for the cleavage with less efficiency as the knock-out of furin does not totally abolish SARS-CoV-2 infection and replication [43]. The S1 subunit, especially the RBD, exhibits high structural flexibility, shaping an active “up” or inactive “down” states by conformational change to modulate the accessibility to the ACE2 receptor [44]. Once the S1 domain of the active state interacts with the ACE2 receptor, another cleavage essential for the activation of SARS-CoV-2 in post fusion stage is the cleavage at the S2′ site, which can be achieved by the action of transmembrane serine protease 2 (TMPRSS2) on the cell surface or by cathepsin L in the endosomal compartment [45,46]. The cleavage at the S2ʹ site exposes the fusion peptide and the fusion machinery of the S2 subunit to the host cell membrane and further drives their fusion to form a fusion pore in the host cell membrane, facilitating the entry of viral gRNA into the host cell [44,47]. It has been shown that ACE2 undergoes SUMOylation (conjugation with small ubiquitin-like modifier 3) at the lysin (K) 187 residue that compromises its K48-ubiquitination, in turn, suppressing ACE2 protein degradation mediated by the TOLLIP (Toll interacting protein) cargo receptor in the autophagic degradation system. As such, inhibition of ACE2 SUMOylation to destabilize ACE2 can be an attractive approach to combat SARS-CoV-2 [48].

### 2.4. Accessory Proteins

Accessory proteins are considered to be dispensable for SARS-CoV-2 replication. The overall functions of the accessory proteins include dysregulation of host defense response and physiological activities and induction of apoptosis of the host cell, thus contributing to the pathogenicity of SARS-CoV-2 virus [49]. ORF3a and ORF7a demonstrate potent antagonist activity against autophagy [50], and ORF3a shows proapoptotic activity by activation of caspase-3 [51]. The contribution of ORF3a to the pathogenicity of SARS-CoV-2 is also supported by the observation that the deletion of ORF3a can reduce the cytokine storm in the host cell, reflected by the decreased ratio of IL6/IL10 [52]. ORF6 and ORF8 can suppress the host defense response by inhibiting the promoter activity of NF-κB transcription factor and its responsive gene of type I interferon IFN-α [53]. Similarly, ORF7b can induce the expression of type I interferon IFN-β, tumor necrosis factor TNF-α, and interleukin IL-6, and stimulate caspase-mediated apoptosis [54]. ORF9b is localized to mitochondria in the SARS-CoV-2 infected cell and can interact with mitochondrial importer receptor Tom70 of the translocase of mitochondrial outer membrane (TOM) complex, potentially interacting with its preprotein substrate binding site [55,56]. In addition, the overexpression of ORF9b and SARS-CoV-2 infection lead to reduced expression of Tom70, thus affecting the functions of Tom70 in mediating translocation of preprotein from cytosol to mitochondria and in recruiting antiviral proteins in mitochondria antiviral signaling (MAVS) [57].

### 2.5. Variants and Mutations

Although there is a proofreading mechanism in SARS-CoV-2 during its genome replication for the sake of genome conservation, mutations still occurred during the pandemic and led to the generation of lineages and variants. Especially, mutations in ExoN of nsp14 apparently can cause a higher mutation load [58], and deletion mutations can escape the correction by proofreading activity [59]. The World Health Organization has assigned simple labels for the SARS-CoV-2 variants using letters of the Greek alphabet (www.who.int). Since its initial burst in late 2019, the variants of SARS-CoV-2 considered variants of concern (VOC) include Alpha, Beta, Gamma, Delta, and Omicron, and each contains multiple PANGO lineages (cov-lineages.org). Omicron is the current variant circulating around the world, and includes BA1, BA2, BA3, BA4, and BA5 sub-lineages. The mutations can occur in the spike protein and other proteins, and the mutations in the spike protein may enhance its cleavage by furin, thus potentially facilitating viral cell entry, increasing viral transmissibility, and resulting in waning antibody efficacy [60]. The mutations not including the insertions and deletions in the spike protein of the Omicron variant with at least 75% prevalence include 20 substitutions, with K417N, S477N, N501Y, P681H being the mutations of interest [61]. The K417N, S477N, N501Y mutations in the receptor binding domain of the S protein can strengthen its interaction with the ACE2 receptor [62], and the P681H mutation resides in the spike S1/S2 cleavage site that potentially increases its cleavage [63]. However, these mutations are not unique to the Omicron variant.

## 3. Threat to Marine Animals

It has been reported that SARS-CoV-2 can be transmitted from humans to pet animals, zoo and farm animals, and wild animals [64]. According to the CDC report, companion animals such as cats, dogs, hamsters, and ferrets, as well as wildlife such as mink, white-tailed deer, and mule deer, and animals in zoos and sanctuaries, such as lions and tigers, can catch SARS-CoV-2 virus, with more than 400 confirmed cases in total in the USA to date. The report from the World Organization for Animal Health (WOAH) released on 31 July 2022 indicates that a total of 679 outbreaks have been reported worldwide [65]. The transmission potential has raised the concern that SARS-CoV-2 might be transmitted to more wild animals with possible susceptibility, and thus precautionary measures should be in practice for outdoor activities such as wildlife research [66]. Genomic sequencing analysis of SARS-CoV-2 isolated from mink and white-tailed deer suggests that no significant mutations or accelerated mutation rate has occurred during the spillover from human to animal for viral adaption to the animal host, implying the nature of a general mammalian virus of SARS-CoV-2 [67].

Under the pandemic circumstances, human body fluids, feces, and contaminants, through close contact containing SARS-CoV-2 particles, may enter the sewage system, and identification of the viral particles provides an efficient early surveillance of the emergence of new SARS-CoV-2 variants and an earlier indicator of its rising incidence in the community [68,69]. The contaminated wastewater may eventually flow into the sea without sufficient treatment, or spill over to the saltwater environment. With an attempt to remove SARS-CoV-2 viral particles from wastewater, an algae-based microrobot was designed using *Chlamydomonas reinhardtii* as the self-driven matrix and fusing ACE2 protein to the algal cell surface, which can effectively adsorb the spike protein and SARS-CoV-2 pseudovirus in the tested aqueous media [70].

Shellfish are used as sentinels to monitor the potential contamination of SARS-CoV-2 in marine coastal areas; no apparent contamination of SARS-Co-2 was detected in the French shores in the summer of 2020 [71]. However, in another similar study, the SARS-CoV-2 RNA was detected in estuarine sediments and in bivalve molluscan species from a natural clam bank in Spain [72]. By using PMAxxx DNA modifier dye, which can only permeate dead cells and covalently binds to nucleic acid after photoactivation, to differentiate free RNA or damaged virion from intact encapsidated viral RNA, the PMAxxx-triton viability RT-PCR assay demonstrated that no infectious viral particles were detected even though the SARS-CoV-2 RNA was detected [72]. The absence or under the detection limit of the viable SARS-CoV-2 virion might be partially explained by the fact the marine high pH and salinity can affect the infectivity and integrity of SARS-CoV-2 virus [73].

**Figure 4:**
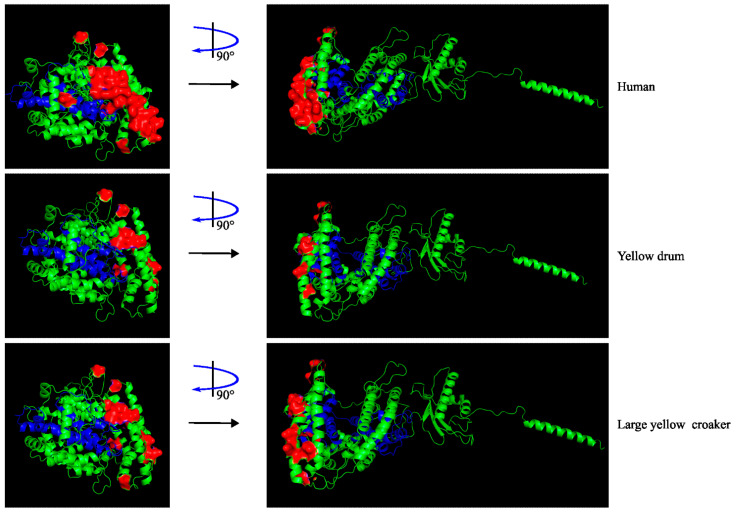
Three-dimensional structures of ACE2 protein in huamn (GenBank No. NP_S.1) and marine yellow drum (GenBank No. KAG8001285.1) and large yellow croaker fish (GenBank No. XP_010730146.1). The tertiary structure oSSf ACE2 protein were predicted with SWISS-MODEL workspace (swissmodel.expasy.org) and visualized by Visual Molecular Dynamics 1.9.4a51 and Pymol. Blue represents the active sites of ACE2 (346–517aa residues), and red represents the 25 spike-interacting key residues and their substitutes.

Meanwhile, the possibility of marine animals harboring or getting infected with SARS-CoV-2 may exist. A novel nidovirus, Pacific salmon nidovirus (PsNV), which is distantly related to SARS-CoV-2, has been detected in wild keystone salmon of the northeastern Pacific with high abundance in the gill tissue, potentially causing declines in their population [74]. Additionally, air-breathing marine animals such otters and dolphins may exposure to SARS-CoV-2 or act as intermediate hosts of SARS-CoV-2, posing the risk of zoonotic COVID-19 disease [75]. Concerns over the spread of SARS-CoV-2 to marine wildlife in Antarctica have also been discussed, exploring the transmission potentials by human activities, human-to-animal-to-animal route, wastewater, and marine animal migration [76]. Generally, the binding affinity of SARS-CoV-2 spike protein to the ACE2 receptor of animals is a factor in the success of viral infection and the determination of susceptibility of the host to SARS-CoV-2, while some other factors play a role as well. The cold temperature of the Antarctic ocean may favor the viability of SARS-CoV-2 for an extended period of time, and the predicted high binding affinity of ACE2 of Antarctic minke whales and killer whales, and the medium affinity of the ACE2 of sperm whales, suggest the infection potentials of these Antarctic mammals by SARS-CoV-2 [76,77]. In addition, other cetacean species including the bottlenose dolphin, Pacific white-sided dolphin, baiji, beluga whale, long-finned pilot whale, and vaquita, are predicted to be highly susceptible to SARS-CoV-2 infection after computational analysis of their ACE2 binding affinity to the viral spike protein: baiji and vaquita are on the brink of extinction [77,78]. Additionally, sea otters of *Fissipedia* and Hawaiian monk seal of *Pinnipedia* are endangered species with high susceptibility to SARS-CoV-2 [78]. In a similar study by analysis of the 25 amino acids of ACE2 interacting with SARS-CoV-2 spike protein, the marine mammals living in Italian coastal waters are supposed to have medium to high susceptibility to SARS-CoV-2 infection, and the immunohistochemistry for ACE2 protein distribution in lung tissues of cetacean species suggests that ACE2 is expressed in alveolar and bronchial epithelium, supporting viral infection potentials [79]. On the other hand, fish such as zebrafish, Nile tilapia, large yellow croaker, and rainbow trout all are predicted to have very low susceptibility to SARS-CoV-2 [77], and no fish cell lines or HeLa cells transfected with ACE2 orthologs of some fish species have shown any infection possibility by SARS-CoV-2 [80]. A comparison of the computed three-dimensional structures of hACE2 and ACE2 proteins of *Nibea albiflora* (yellow drum) and *Larimichthys crocea* (large yellow croaker) marine fishes shows the differential spatial distribution of the spike-interacting residues (Figure 4), which may account for the low affinity of marine fish ACE2 proteins to the spike protein of SARS-CoV-2.

## 4. Antiviral Activity against SARS-CoV-2 in Marine Resources

Since the outbreak of COVID-19, tremendous endeavors have been undertaken to find effective antiviral strategies against SARS-CoV-2 infection. The development and distribution of COVID-19 (SARS-CoV-2) vaccines have greatly helped people alleviate the risk of getting seriously ill if contracting SARS-CoV-2. To date, there are four COVID-19 vaccines that have been authorized by the Food and Drug Administration (FDA) in the US, including Pfizer-BioNTech, Moderna, Johnson & Johnson’s Janssen, and the most recently approved Novavax, with notable effectiveness [81]. Some new vaccines are also under development, such as the plant-based virus-like particle (CoVLP) vaccine by adjuvanting the purified modified SARS-CoV-2 spike protein expressed in *Nicotiana benthamiana* with AS03 adjuvant, which has demonstrated notable cross-reactivity against different SARS-CoV-2 variants [82]. However, with the everchanging genomic information of SARS-CoV-2 and the waning of immunity, breakthrough infections of SARS-CoV-2 have occurred in many cases in vaccinated people [83]. In addition, some antiviral drugs, monoclonal antibodies, and immune-modulators have been developed as COVID-19 therapeutics for emergency use. Veklury (remdesivir) and Olumiant (baricitinib) are so far the two FDA-approved drugs for treatment of COVID-19 in the US. Remdesivir is a prodrug, an adenosine nucleotide analogue, which undergoes intracellular phosphorylation to be accommodated by the RdRp of SARS-CoV-2 to inhibit the viral RNA synthesis process [84]. Baricitinib is a Janus kinase (JAK) inhibitor with anti-inflammatory property, demonstrating activities in reducing receptor-mediated viral endocytosis and suppressing cytokine storm, and has been repurposed as a medication to treat hospitalized adults with moderate to severe COVID-19 [85,86]. In the meantime, scientists have devoted tremendous effort to identifying new compounds targeting SARS-CoV-2 and the post-infection immune system to combat viral infection and the disease through in silico, in vitro, and in vivo approaches [87,88]. Natural products with antimicrobial and antiviral properties are of great interest to scientists to explore their efficiency in combating COVID-19 [89]. It has been known for many decades that marine products serve as a seemingly limitless bio-resource for combating pathogenic microbes and cancers [90]. Studies on marine-derived antiviral compounds have discovered a wide collection of bioactive molecules, with different targets of components shaping SARS-CoV-2 successful infection and proliferation (Table 2), aiming to find alternative pharmaceuticals with enhanced specificity to SARS-CoV-2 and reduced side effects on the human body. Here some extensively studied compounds are further discussed with more details; for brevity, not all the functions of the compounds are mentioned.

### 4.1. Targeting Viral Recognition and Interaction

#### 4.1.1. Sulfated Polysaccharides

The entry of SARS-CoV-2 is initialized largely by the interaction between the viral spike protein and the host ACE2 receptor through attraction by electrostatic forces, in which the RBD of spike protein is dominantly positively charged while the ACE2 has a negatively charged surface [112]. Sulfated polysaccharides are highly diverse and abundant in the ocean, especially in macroalgae and some marine animals harboring polyanion of sulfate ions, which have been demonstrated to be effective against SARS-CoV-2 entry into host cells [91]. Three types of marine sulfated polysaccharides including sea cucumber sulfated polysaccharide (SCSP), fucoidan from brown algae, and ι-carrageenan from red algae have been shown to be capable of binding the spike protein of SARS-CoV-2, with SCSP exhibiting the strongest inhibitory effect [113]. A nasal spray containing ι- and κ-carrageenan, and an oral spray containing ι-carrageenan, showed anti-SARS-CoV-2 activity by preventing the attachment of viral particles to, and its entry into, TMPRSS2-expressing Vero E6 cells, while it did not apparently affect the host cell viability [114]. In addition, the λ-carrageenan from marine red algae was shown to be able to inhibit the entry of SARS-CoV-2 spike-pseudotyped virus and the infectious SARS-CoV-2 into Vero E6 cells, interfering with the spike protein-associated entry step [115].

#### 4.1.2. Inorganic Polyphosphates

Inorganic polyphosphate (polyP) is another negatively charged polymer that has shown promising antiviral effect against SARS-CoV-2. polyPs with varying lengths are ubiquitously distributed in all living organisms, and involved in many physiological functions [116]. The enriched accumulation of polyP has been found in some marine bacteria and marine sponges [117,118]. A polyP with as short as 3 phosphate (P_i_) units (polyP_3_) can significantly inhibit the binding of RBD of SARS-CoV-2 to ACE2, potentially through negatively charged P_i_ units of polyP interacting with the positively charged residues of Arg, Lys, and His of RBD through electrostatic interaction, especially since chemical modification of Arg residues with increased reactivity can enhance the inhibitory efficiency of polyP on RBD binding to ACE2 [92,93]. Besides its ability to bind RBD of SARS-CoV-2, long-chain polyP (polyP_120_) can also bind ACE2 through the interaction with its positively charged residues His_378_, His_401_, Arg_393_, and Arg_514_, leading to the proteasome-mediated degradation of ACE2 [107]. Furthermore, the polymer polyP_40_ showed activating effects on the expression of MUC1 and MUC5AC, which are membrane-tethered mucin and the gel-forming secreted mucin, respectively, after the polyP in a collagen hydrogel-mucin environment was attached to human alveolar basal epithelial A549 cells, preventing the invasion of SARS-CoV-2 in the epithelium of the airway and lung [109]. The secretion of mucin can benefit from the generation of ATP through the hydrolysis of polyP by ALP (alkaline phosphatase) and the phosphorylation of ADP by ADK (adenylate kinase) [109,119], and quercetin antioxidant and the synthetic anti-inflammatory dexamethasone in caged nanoparticles with polyP can enhance the effect of polyP on mucin production [119].

Additionally, polyP is found in dense granules of platelets, which release polyP after activation, subsequently triggering the initiation of blood clotting and the liberation of inflammatory mediators [120]. Later studies indicated the activity of polyP in triggering blood clotting varies depending on the length of the polymer [121], and a conflicting result was also reported in which the synthetic polyP can inhibit blood clotting by reducing the levels of Ca^2+^ and thromboxane affecting platelet aggregation [122]. It has been shown that severe symptomatic COVID-19 patients have hyperactivated platelets and a noted drop in the count of platelets, which induce local thrombus formation and a systemic coagulation defect resulting in serious and even fatal consequence [123,124]. These observations suggest that exogenous supplementation of polyP might be an option, as the high consumption of platelets in COVID-19 patients with thrombocytopenia, and a unit length of P_50_ was suggested, while further clinical studies are required [125].

#### 4.1.3. Cyanobacteria Molecules

As well as their richness in sulfated polysaccharides, cyanobacteria possess other bioactive compounds such as pigments and amino acids. Four bioactive molecules (phycoerythrobilin, phycocyanobilin, phycourobilin, and folic acid) were identified, by molecular docking assays, in the microalgae *Arthrospira* with high binding affinity to the RBD region of SARS-CoV-2 spike protein and good bioavailability [94]. Additionally, through molecular docking, ADME (absorption, distribution, metabolism, excretion), and cell toxicity analysis for isolation of natural inhibitors against ACE2 from cyanobacteria bioactive compounds, mycosporine-glycine-valine and shinorine demonstrated low binding energy to ACE2, high solubility, and free of toxicity, providing strong potentials for antiviral drug development [108]. Another important group of antiviral molecules is the mannose-specific lectins, which are found in cyanobacteria and red and green algae [95]. SARS-CoV-2 can use the C-type lectin receptors such as DC-SIGN, L-SIGN and the sialic acid-binding immunoglobulin-like lectin 1 (SIGLEC1) as attachment receptors to facilitate its presentation to ACE2 receptor for viral trans infection [126]. The mannose-specific lectins, such as cyanovirin from cyanobacteria *Nostoc ellipsosporum* and griffithsin from red algae *Griffithsia* sp. can recognize the N-glycosylated spike protein of SARS-CoV-2 with high-mannose glycans, inhibiting SARS-CoV-2 infection [95].

### 4.2. Targeting Viral Replication

#### 4.2.1. Polyphenols against Mpro

Phlorotannins are secondary metabolites in brown algae, and are oligomers of phloroglucinol. Molecular docking analysis predicted that eckol and trifucol with negative higher binding energy (lower binding energy) to 3CLpro could be good inhibitors of Mpro [96]. By computational modeling to virtually screen a Marine Natural Product (MNP) library for the interaction with Mpro, 17 compounds out of 180 selected molecular after initial pharmacophore filtering stood out as the best candidates showing interaction with Mpro, and phlorotannins represent a major group among these compounds [97]. Further docking studies suggested that 8,8′-bieckol, 6,6′-bieckol, and dieckol of phlorotannins identified in the brown algae *Ecklonia cava* were the most active inhibitors against Mpro. In addition, some flavonoids, including apigenin-7-O-neohesperidoside, luteolin-7-rutinoside, and resinoside were also listed as promising inhibitors of Mpro [97].

#### 4.2.2. Alkaloids against PLpro and Mpro

Alkaloids are a large group of structurally diverse natural compounds, that contain at least one nitrogen atom, with great potential for drug development to treat diseases and mental disorders [127]. Marine sponges provide an abundant resource of bioactive alkaloid compounds [128]. Through molecular docking and molecular dynamics simulation studies to screen polycyclic guanidine alkaloid compounds in marine sponge *Monanchora* n. sp. targeting SARS-CoV-2 Mpro and other proteins, two compounds crambescidin 786 and crambescidin 826, showed high binding affinity to the enzyme pockets of Mpro with very low toxicity and high bioavailability, rendering them promising candidates as anti-SARS-CoV-2 drugs [98]. By employing a similar approach to probe marine compounds targeting Mpro of SARS-CoV-2 as potential inhibitors, an alkaloid compound fistularin 3 (also known as isofistularin-3; PubChem CID 159041) isolated from marine sponges of the *Aplysinidae* family was identified as the most potent candidate with strong bonding with the amino acid residues in the active site of Mpro [99]. Another virtual screen for anti-SARS-CoV-2 secondary metabolites from marine and terrestrial fungi identified three fumiquinazoline marine alkaloids scedapin, norquinadoline A, and quinadoline B that showed high affinity to the putative binding site of PLpro [100]. Another marine algae-derived alkaloid caulerpin was isolated after virtual screen of 10 bioactive natural compounds for the anti-SARS-CoV-2 potentials targeting Mpro, demonstrating the highest binding affinity to Mpro among the tested compounds [101]. The high negative free binding energy between caulerpin molecule and the modelled Mpro suggests caulerpin could be an effective antiviral drug against SARS-CoV-2.

#### 4.2.3. Plitidepsin against Host Factor eEF1A

Plitidepsin, also known as dehydrodidemnin B, is a cyclic depsipeptide. It is a marine-derived compound extracted from Mediterranean tunicate *Aplidium albicans*, which has shown anticancer activity potentially targeting the eukaryotic elongation factor 1A2 (eEF1A2) [110]. Evaluation of the antiviral effect of plitidepsin as a repurposed drug against SARS-CoV-2 showed that plitidepsin can significantly inhibit SARS-CoV-2 replication post viral entry in Vero E6 cells and hACE2-293T cells with a substantially lower IC_90_ compared to remdesivir, while cytostatic impact on cell proliferation was observed [111]. The antiviral and antiproliferative actions of plitidepsin are mediated by its inhibitory activity on eEF1A by introduction of the mutated version eEF1A-A399V into hACE2-293T imparts the cells resistance to plitidepsin treatment, and the refractory effects can be rescued by transfection with the wild-type eEF1A [111]. The antiviral activity of plitidepsin was shown by its marked inhibition of SARS-CoV-2 viral genomic RNA replication and sub-genomic N RNA synthesis and the N protein expression after initial viral infection, with a stronger effect than remdesivir. In vivo data obtained from the infection of hACE2-sensitized or hACE2-transgenic mouse further supported antiviral effect of plitidepsin against SARS-CoV-2 [111]. Further investigation of the antiviral activity of plitidepsin on SARS-CoV-2 by transmission electron microscopy and immunohistochemistry was performed to examine its effect on viral replication [129]. It was evident that plitidepsin treatment induced the disappearance of DMVs structure for viral genome replication, and the absence of viral particle distribution in single-membrane vesicles, in the large vacuole, and on the extracellular side of the plasma membrane in Vero E6 cells at 24–D48 h post infection. In addition, the viral N protein and dsRNA were not detected by immunostaining in plitidepsin-treated Vero E6 cells at 48 h post infection [129]. A more recent study showed that plitidepsin can virtually bind to the main protease of SARS-CoV-2 and inhibit its activity [130].

## 5. Conclusions

This review aimed to provide an update on our understanding of SARS-CoV-2 genome composition and viral components for virion replication and assembly, and to present an overview of SARS-CoV-2 infection mechanism in host cells. The viral genome largely encodes structural proteins of spike, envelop, membrane, and nucleocapsid proteins, and some nonstructural and accessory proteins facilitating SARS-CoV-2 proliferation and infection. Given the complexity of SARS-CoV-2 genome composition, the translation and function of the molecular fragments are not fully understood yet and more in vivo studies are required to explore the unknowns. In addition, the potential of spreading SARS-CoV-2 to marine mammals has been reviewed largely based on the compatibility of host ACE2 receptor to the spike of SARS-CoV-2 while other entry routes may exist, at least in an auxiliary manner. Precaution and monitoring are necessary to avoid spillover to marine animals, although there has been no report about infection of marine mammals by SARS-CoV-2 yet. Antiviral drug development has driven enormous efforts in discovering natural products such as marine metabolites with anti-SARS-CoV-2 properties through in silico, in vitro, and in vivo approaches. One compound may target more than one protein component of SARS-CoV-2 to mitigate its infection. The most promising bioactive compounds, such as inorganic polyphosphates and plitidepsin, are to undergo comprehensive and thorough evaluation for their eventual application in clinical treatment.

## Figures and Tables

**Table 1:** The basic description of the nonstructural proteins based on annotation of the GenBank ID NC_045512.2. and with reference to a recent review on SARS-CoV-2 genomic structure [29].

Protein	Amino Acids	Function
nsp1	180	Leader protein
nsp2	638	Zinc-finger protein
nsp3	1945	Papain-like proteinase (PLpro)
nsp4	500	Tetra spanning transmembrane protein
nsp5	306	3C-like proteinase (3CLpro) or main protease (Mpro)
nsp6	290	Transmembrane domain-containing protein
nsp7	353	A component of primase complex with nsp8 and nsp12
nsp8	198	A component of primase complex with nsp7 and nsp12
nsp9	113	ssRNA-binding protein
nsp10	139	Interacting with nsp14 and nsp16
nsp11	13	n.d.(not defined)
nsp12	932	RNA-dependent RNA polymerase (RdRp)
nsp13	601	Helicase
nsp14	527	N^7^-Methyltransferase and 3′-5′ exonuclease (ExoN)
nsp15	346	Uridine-specific endoribonuclease
nsp16	298	2′-O-ribose Methyltransferase

**Table 2:** Marine-derived compounds as potent inhibitors of SARS-CoV-2.

Target	Marine Compound	Reference
Viral spike protein	Sulfated polysaccharides	[91]
	Inorganic polyphosphates	[92,93]
	Phycobilins	[94]
	Mannose-specific lectins	[95]
Mpro and/or PLpro	Polyphenols	[96,97]
	Alkaloids	[98,99,100,101]
	Phycobilins	[102]
	Coumarin derivatives	[103]
	Naphthalene derivatives	[104]
RdRp	Nucleoside analogues	[105]
TMPRSS2	Watasenia preluciferyl β-D- glucopyranosiduronic acid	[106]
hACE2	Inorganic polyphosphates	[107]
	Mycosporin-like amino acids	[108]
Immune system	Inorganic polyphosphates	[107,109]
Host eEF1A	Plitidepsin	[110,111]

## Data Availability

Not applicable.
